# The gonadal niche safeguards human fetal germline cell development following maternal SARS-CoV-2 infection

**DOI:** 10.1016/j.xcrm.2024.101515

**Published:** 2024-04-16

**Authors:** Shijun Shen, Mengting Wang, Xiaocui Li, Beiying Wang, Wei Hong, Wei Li, Ben Xu, Zhenxiang Guo, Ruichen Han, Shanru Yi, Zhiping Wu, Xiaoying He, Liping Wang, Qianshu Zhu, Guang Yang, Hong Wang, Qiaolin Deng, Jiayu Chen, Shaorong Gao, Cizhong Jiang, Rui Gao

**Affiliations:** 1 Clinical and Translational Research Center of Shanghai First Maternity and Infant Hospital, Shanghai Key Laboratory of Signaling and Disease Research, School of Life Sciences and Technology, Tongji University, Shanghai 200092, China; 2 Key Laboratory of Spine and Spinal Cord Injury Repair and Regeneration of the Ministry of Education, Orthopedic Department of Tongji Hospital, Tongji University, Shanghai 200065, China; 3 Shanghai Key Laboratory of Maternal Fetal Medicine, Shanghai Institute of Maternal-Fetal Medicine and Gynecologic Oncology, Shanghai First Maternity and Infant Hospital, School of Medicine, Tongji University, Shanghai 201204, China; 4 Frontier Science Center for Stem Cell Research, Tongji University, Shanghai 200092, China; 5 Department of Physiology and Pharmacology, Biomedicum B5, Karolinska Institutet, Center for Molecular Medicine, Karolinska University Hospital, 17177 Stockholm, Sweden

**Keywords:** germline cell, gonadal niche, human reproduction, pregnancy, SARS-CoV-2, COVID-19, immune response, developing fetus, host defense

## Abstract

During pregnancy, germline development is vital for maintaining the continuation of species. Recent studies have shown increased pregnancy risks in COVID-19 patients at the perinatal stage. However, the potential consequence of infection for reproductive quality in developing fetuses remains unclear. Here, we analyze the transcriptome and DNA methylome of the fetal germline following maternal severe acute respiratory syndrome coronavirus 2 (SARS-CoV-2) infection. We find that infection at early gestational age, a critical period of human primordial germ cell specification and epigenetic reprogramming, trivially affects fetal germ cell (FGC) development. Additionally, FGC-niche communications are not compromised by maternal infection. Strikingly, both general and SARS-CoV-2-specific immune pathways are greatly activated in gonadal niche cells to protect FGCs from maternal infection. Notably, there occurs an “in advance” development tendency in FGCs after maternal infection. Our study provides insights into the impacts of maternal SARS-CoV-2 infection on fetal germline development and serves as potential clinical guidance for future pandemics.

## Highlights

• Analysis of the developing human gonads in the context of COVID-19 pandemic
• Trivial impacts but an “in advance” development trend in FGCs upon maternal infection
• Preserved DNA demethylation pattern in FGCs following maternal infection
• Extensive immune responses in gonadal niche shield FGCs from maternal infection

## Graphical abstract

**Figure:**
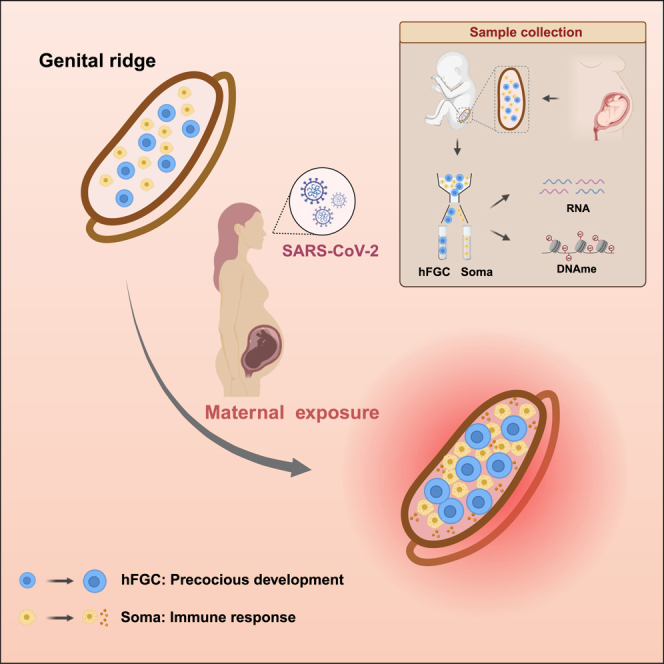

## Teaser

Shen et al. interrogate the transcriptome and DNA methylome of developing human gonads in the context of the COVID-19 pandemic. Trivial impacts but a precocious development trend occur in fetal germ cells (FGCs) following maternal infection. Extensive immune responses are activated in the gonadal microenvironment to preserve the integrity of FGC development.

## Introduction

The global spread of severe acute respiratory syndrome coronavirus 2 (SARS-CoV-2) has posed a major threat to human public health.^1^^,^^2^ People infected with such a new coronavirus, in general, suffer from physical disorders and mental distress, including pregnant women.^3^ Mounting evidence has indicated that such infection during the late trimester of pregnancy could significantly increase the rates of maternal and neonatal complications, even resulting in mortality in some cases.^4^^,^^5^^,^^6^^,^^7^ Notably, the wide distribution of the SARS-CoV-2 receptor ACE2 and the protease TMPRSS2 in human trophectoderm cells of pre-gastrulation embryos and the maternal-fetal interface raises intensive concerns.^8^^,^^9^ Such findings further suggested that SARS-CoV-2 infection might impair prenatal health and successful pregnancy, especially during the first trimester.

A particularly important event during early gestation is the emergence of the germ cell population.^10^ Germline development is an integral part of the life cycle, culminating in the generation of functional gametes, which faithfully transmit genetic and epigenetic information from one generation to the next.^11^^,^^12^^,^^13^ It has emerged that specification, migration, localization to genital ridges, and sex differentiation of the germ cells occur in human embryos during the first trimester of pregnancy.^14^ Recently, we and other groups have shown that epigenetic and transcriptional reprogramming of fetal germ cells (FGCs) is important for resetting the developmental potential, erasing epigenetic memory, and establishing unipotency in human germ cells.^15^^,^^16^^,^^17^^,^^18^^,^^19^^,^^20^^,^^21^^,^^22^ However, it remains unclear whether the human germline, when exposed to external threats, can still have normal progression. Although previous case studies of clinical cohorts have documented the adverse neonatal outcomes of maternal SARS-CoV-2 infection,^4^^,^^5^^,^^6^^,^^7^ questions concerning the potential consequences of such infection for reproduction quality in the developing fetus were still largely unaddressed. Thus, understanding the impact of SARS-CoV-2 on human germline cell development is particularly important for assessing the broader implications of the pandemic on the health and safety of pregnant women and newborns, as it defines the final reproductive outcomes, which is crucial for proper maintenance of the species.

To address this concern, we investigated the cellular and molecular effects of SARS-CoV-2 linking maternal infection to fetal quality, especially prenatal germline development. We found that infection at the early gestational age, a critical period for human germ cell specification and epigenetic reprogramming, did not obviously impair germline outcomes, including the proper determination and maintenance of the germ cell fate, preserved genome-wide rapid demethylation, erased DNA methylation in the imprinted regions, X chromosome reactivation, and epigenetic repression of retrotransposons. However, an “in advance” developmental status in FGCs was noticed. More strikingly, we found that SARS-CoV-2 infection during pregnancy is primarily associated with extensive immune responses in the embryonic gonadal microenvironment. Such effective adaptive defenses in the developing fetus help to preserve the integrity of FGC development. This, in turn, guarantees the transfer of immune memory and genetic information to the next generation following maternal infection. Our study provides insights into individuals of reproductive age and presents a significant advancement in understanding the epigenetic and transcriptional programs of the human germline following maternal perturbations.

## Results

### Clinical characteristics of the study cohort

**Figure 1:**
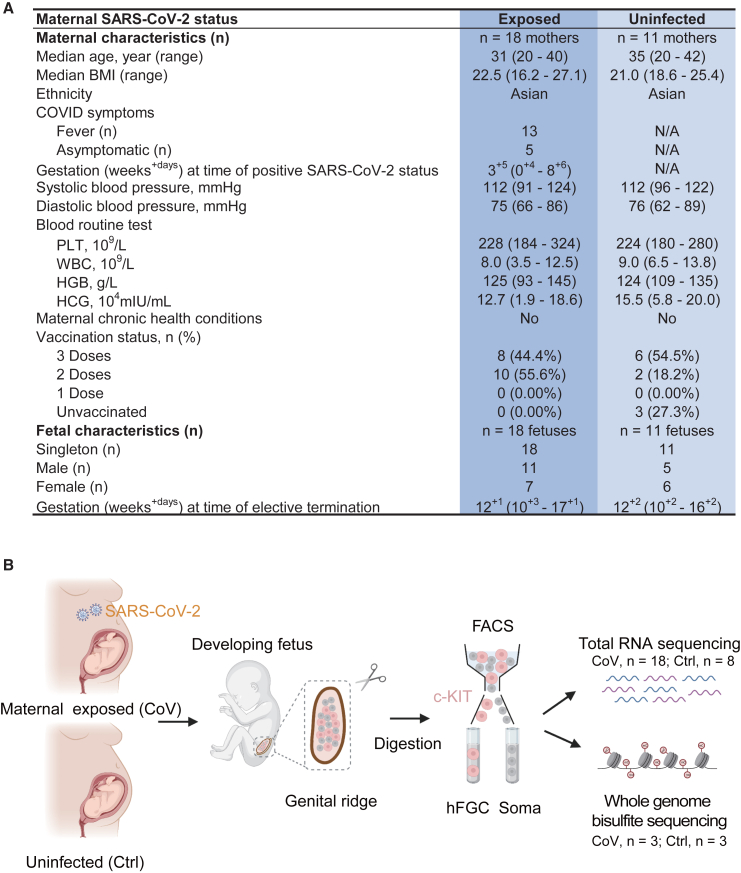
Research overview of developing human gonads in the context of the COVID-19 pandemic (A) Maternal and fetal clinical characteristics of collected samples. N/A, not available; BMI, body mass index; PLT, platelet count; WBC, white blood cell count; HGB, hemoglobin; HCG, human chorionic gonadotropin. (B) Schematic of the generation of transcriptomics and epigenomics data of fetal germ cells (FGCs) and gonadal somatic cells (soma) from the developing fetus upon maternal exposure to SARS-CoV-2 or no exposure. CoV, maternal exposed group (SARS-CoV-2). Ctrl, uninfected group. The plot was created by BioRender. See also Figure S1.

A total of 29 women who underwent elective termination of pregnancy were enrolled in our study. All of these pregnant individuals completed a COVID-19 infection questionnaire. The clinical characteristics of the study population are displayed in Figure 1A and Table 1. The median gestational age at the time of positive SARS-CoV-2 status was less than 4 weeks. No obvious pregnancy-related complications, including gestational diabetes, hypertensive disorder of pregnancy, or placenta previa, were detected in these individuals. Embryo samples ranging from 10–17 weeks after gestation were collected upon admission to systematically investigate the potential impact of maternal SARS-CoV-2 infection on human FGC development and the regulatory relationships between FGCs and their neighboring niche cells. The exposed group (7 female and 11 male embryos) was derived from pregnant women who tested positive for COVID-19 during the first trimester of pregnancy but recovered at termination. Meanwhile, the uninfected group (6 female and 5 male embryos) from non-infected pregnant women was set as a control.

**Table 1:** Clinical characteristics of collected human samples

Patient ID	Maternal SARS-CoV-2 status	Gender	Gestational age at infection	Gestational age at termination	Maternal age (years)	Fever	Fever temperature (°C)	Fever duration (days)	Vaccination	Vaccination dose	Height (cm)	Weight (kg)	BMI	Diastolic blood pressure (mm Hg)	Systolic blood pressure (mm Hg)	PLT (10^9^/L)	WBC (10^9^/L)	HGB (g/L)	HCG (mIU/mL)
P1	maternal exposed (CoV)	female	4 weeks	11 weeks	32	yes	37.8	3	yes	3	174	69.0	22.79	80	108	213	7.28	118	111,592.1
P2	maternal exposed (CoV)	female	3 weeks	11 weeks	32	no	N/A	N/A	yes	2	156	45.0	18.49	86	123	324	8.00	141	68,704.8
P3	maternal exposed (CoV)	female	3 weeks	11 weeks	24	yes	38.0	4	yes	2	160	65.0	25.39	71	109	243	10.52	131	181,543.9
P4	maternal exposed (CoV)	female	4 days	12 weeks	33	no	N/A	N/A	yes	3	155	65.0	27.06	82	107	236	7.38	122	84,276.4
P5	maternal exposed (CoV)	female	5 weeks	13 weeks	37	yes	39.0	3	yes	3	165	60.0	22.04	77	118	239	8.84	122	140,303.6
P6	maternal exposed (CoV)	female	2 weeks	13 weeks	27	yes	38.3	2	yes	3	165	47.5	17.45	67	91	271	6.09	98	154,133.6
P7	maternal exposed (CoV)	female	5 weeks	15 weeks	29	no	N/A	N/A	yes	2	158	60.0	24.03	78	108	221	5.40	118	19,092.7
P8	uninfected (Ctrl)	female	N/A	11 weeks	36	N/A	N/A	N/A	yes	2	158	50.0	20.03	81	120	224	9.72	109	200,000.0
P9	uninfected (Ctrl)	female	N/A	11 weeks	41	N/A	N/A	N/A	yes	3	172	62.0	20.96	76	119	240	9.89	132	N/A
P10	uninfected (Ctrl)	female	N/A	12 weeks	20	N/A	N/A	N/A	yes	3	158	53.0	21.23	83	120	241	7.35	124	174,575.9
P11	uninfected (Ctrl)	female	N/A	14 weeks	25	N/A	N/A	N/A	no	N/A	165	62.4	22.92	89	107	198	10.21	118	136,718.1
P12	uninfected (Ctrl)	female	N/A	16 weeks	25	N/A	N/A	N/A	yes	3	160	65.0	25.39	76	114	238	9.07	121	77,850.1
P13	maternal exposed (CoV)	male	4 weeks	10 weeks	32	yes	40.0	3	yes	2	150	42.0	18.67	66	101	254	7.89	99	180,866.2
P14	maternal exposed (CoV)	male	2 weeks	11 weeks	38	yes	39.4	3	yes	2	150	44.0	19.56	75	114	195	6.39	138	163,319.6
P15	maternal exposed (CoV)	male	4 weeks	11 weeks	21	yes	39.0	4	yes	3	163	60.0	22.58	67	107	224	8.00	118	126,999.5
P16	maternal exposed (CoV)	male	7 weeks	11 weeks	20	yes	38.5	1	yes	2	165	44.0	16.16	83	110	201	6.40	120	97,487.4
P17	maternal exposed (CoV)	male	3 weeks	12 weeks	26	yes	38.5	1	yes	2	155	50.0	20.81	82	122	285	8.41	128	153,622.8
P18	maternal exposed (CoV)	male	2 weeks	12 weeks	28	no	N/A	N/A	yes	3	163	66.0	24.84	70	115	218	8.85	136	186,653.3
P19	maternal exposed (CoV)	male	5 weeks	13 weeks	24	yes	39.0	3	yes	2	168	65.0	23.03	72	114	232	8.26	131	93,570.9
P20	maternal exposed (CoV)	male	5 weeks	14 weeks	30	yes	37.8	2	yes	3	164	54.0	20.08	79	106	213	7.90	145	146,567.5
P21	maternal exposed (CoV)	male	5 weeks	15 weeks	40	yes	38.6	4	yes	3	170	65.0	22.49	74	124	200	12.54	134	100,650.6
P22	maternal exposed (CoV)	male	2 weeks	15 weeks	34	yes	38.5	3	yes	2	163	64.0	24.09	69	121	184	3.46	93	57,492.4
P23	maternal exposed (CoV)	male	9 weeks	17 weeks	33	no	N/A	N/A	yes	2	162	70.0	26.67	76	120	251	8.46	132	N/A
P24	uninfected (Ctrl)	male	N/A	10 weeks	29	N/A	N/A	N/A	no	N/A	164	50.0	18.59	62	103	277	13.83	127	160,176.9
P25	uninfected (Ctrl)	male	N/A	12 weeks	20	N/A	N/A	N/A	yes	2	163	56.0	21.08	73	97	280	9.01	126	137,382.8
P26	uninfected (Ctrl)	male	N/A	12 weeks	35	N/A	N/A	N/A	yes	3	168	56.0	19.84	74	112	199	7.69	113	58,758.5
P27	uninfected (Ctrl)	male	N/A	11 weeks	36	N/A	N/A	N/A	no	N/A	161	65.0	25.10	75	106	196	6.51	135	187,660.0
P28	uninfected (Ctrl)	male	N/A	11 weeks	40	N/A	N/A	N/A	yes	3	160	50.0	19.50	69	96	180	7.88	109	162,633.7
P29	uninfected (Ctrl)	female	N/A	13 weeks	42	N/A	N/A	N/A	yes	3	160	50.0	19.50	80	122	183	6.78	125	150,265.8

N/A, not available; BMI, body mass index; PLT, platelet count; WBC, white blood cell count; HGB, hemoglobin; HCG, human chorionic gonadotropin. Routine blood tests are performed at the time of elective termination.

Then we examined the transcriptomes and DNA methylomes of human FGCs, which were isolated from individual gonads from both exposed and uninfected subjects. This was achieved by using fluorescence-activated cell sorting (FACS) with the surface receptor c-KIT. Concurrently, c-KIT-negative somatic cells were also collected to uncover potential altered interactions between FGCs and their microenvironment triggered by SARS-CoV-2 infection during pregnancy (Figures 1B and S1A–S1C; Table S1).

### Trivial impact on FGC development following maternal SARS-CoV-2 infection

**Figure 2:**
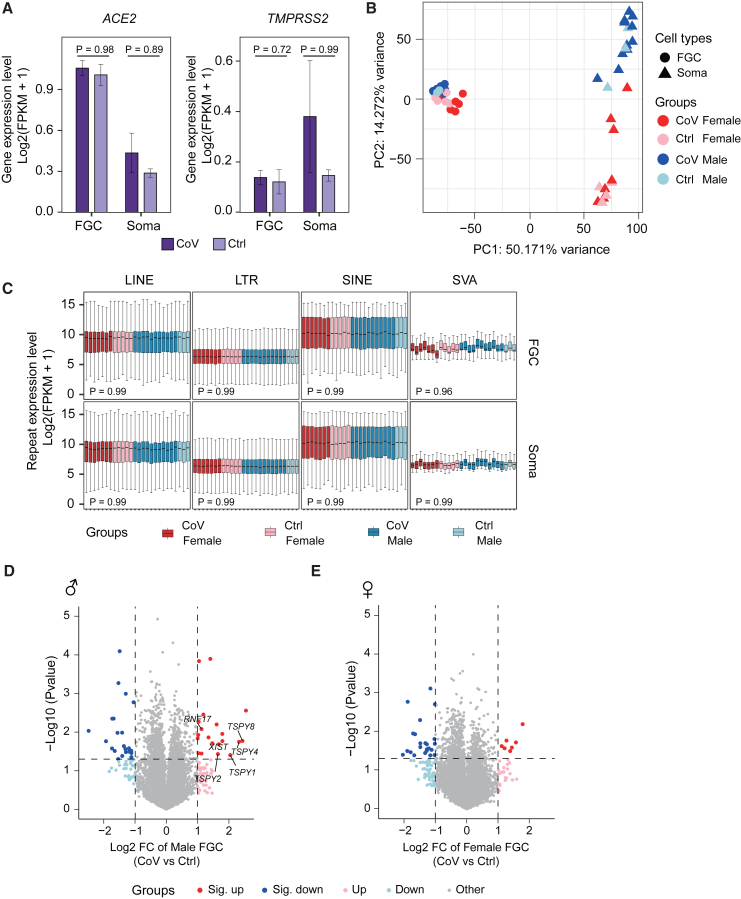
Transcriptome reveals limited impact on human FGC development following maternal SARS-CoV-2 infection (A) Expression level of selected SCARFs (SARS-CoV-2- and coronavirus-associated receptors and factors) in FGCs and gonadal somatic cells. The expression level is represented as log2(FPKM+1). The *p* values were calculated by Wilcoxon rank-sum test. CoV_*n* = 18; Ctrl_*n* = 8. (B) Principal-component analysis (PCA) of the transcriptomes of FGCs and gonadal somatic cells from the CoV and Ctrl groups. Circle, FGC; triangle, gonadal somatic cell; red color scheme, female; blue color scheme, male. The variance of PC1 and PC2 is shown. (C) Expression level of major human repetitive element classes in FGCs and gonadal somatic cells. The expression level is represented as log2(FPKM+1). Wilcoxon rank-sum test was performed to examine the statistical difference between the CoV group and Ctrl group. Red color scheme, female; blue color scheme, male. The boxplots are ordered by gestational age at termination, horizontally in each group. (D and E) Volcano plots of differentially expressed genes (DEGs) identified by CoV group versus Ctrl group in male (D) or female (E) FGCs. The alternation of gene expression is represented as log2 fold change (log2 FC). Dark red and dark blue dots indicate genes significantly upregulated or downregulated in the CoV group, respectively (|log2 FC| > 1, *p* < 0.05; male_sig._up_*n* = 20, male_sig._down_*n* = 23, female_sig._up_*n* = 7, female_sig._down_*n* = 23). Light red and light blue dots indicate genes upregulated or downregulated in the CoV group (|log2 FC| > 1, *p* ≥ 0.05; male_up_*n* = 37, male_down_*n* = 30, female_up_*n* = 20, female_down_*n* = 50). Gray dots indicate other genes. The *p* values were calculated by Student’s t test. CoV_Male_*n* = 11, Ctrl_Male_*n* = 3, CoV_Female_*n* = 7, Ctrl_Female_*n* = 5. See also Figures S2 and S3.

We first confirmed the expression of SARS-CoV-2- and coronavirus-associated receptors and factors (SCARFs)^23^ in the FGCs and their neighboring niche cells (Figures 2A and S2A), which provide a route for SARS-CoV-2 infection. Careful examination revealed no significant difference in SCARF gene expression in FGCs after maternal SARS-CoV-2 infection, and the same in the somatic cells, except for two genes. However, there existed the trend that SCARF gene expression in the somatic cells was higher after maternal infection (Figures 2A and S2A). This suggested that SARS-CoV-2 infection might increase SCARF gene expression in the gonadal somatic cells but not in FGCs. Therefore, the somatic cells became more susceptible to SARS-CoV-2.

To evaluate the overall impact of SARS-CoV-2 infection on the embryonic gonads, we performed principal-component analysis (PCA) of the transcriptomes and obtained two completely separate groups, germline cells and somatic cells, regardless of the infection status. Besides, gender differences could also be distinguished in the FGCs (Figure 2B). Of note, male and female somatic cells showed distinct gene expression patterns (Figure 2B). This is partially due to the different sex-specific gene expression. For example, *KRT19* and *FOXL2* were expressed in female somatic cells to discriminate pre-granulosa cells,^24^ while *SOX9* and *DMRT1* guide common progenitors to early male somatic lineages^24^^,^^25^ (Figures S2B and S2C; Table S2). Despite the potential fusion of SARS-CoV-2 RNA with retrotransposon-identical transcripts, especially long interspersed nuclear elements (LINEs) and short interspersed nuclear elements (SINEs),^26^^,^^27^ we did not detect a prominent change in the expression of transposable elements (TEs) in FGCs between the exposed group and the uninfected group (Figure 2C). Meanwhile, we identified only 43 and 30 significantly differentially expressed genes (DEGs) in male and female FGCs, respectively, upon maternal exposure to SARS-CoV-2 (Figures 2D and 2E; Table S3). Taken together, infection status only induces a trivial impact on germ cell development outcomes, especially regarding the major differences in both gender specificity and functional significance of the cell types.

### Precocious development tendency in human FGCs upon maternal exposure to SARS-CoV-2

**Figure 3:**
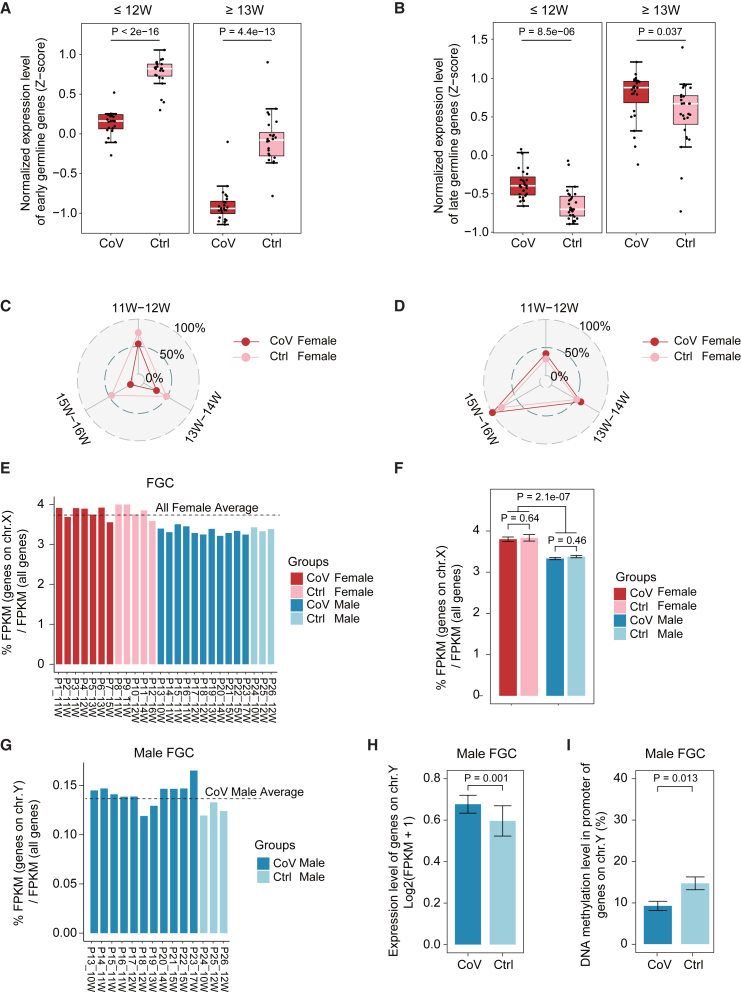
Precocious development tendency in human FGCs upon maternal exposure to SARS-CoV-2 (A and B) Boxplots showing integrated normalized expression levels of early (A) and late (B) germline marker genes in female FGCs (≤12 weeks, CoV_*n* = 4, Ctrl_*n* = 3) and female FGCs (≥13 weeks, CoV_*n* = 3, Ctrl_*n* = 2). The expression level was *Z* scored. Student’s t test was performed to examine the statistical difference between the CoV group and Ctrl group. Week (W), gestational week at termination. (C and D) Radar plots showing the average expression levels of the germline-specific marker genes of female FGCs. (C) The average expression levels of the early germline-specific marker genes of female FGCs in (A) in the CoV group are lower than in the Ctrl group among three developmental stages (11–12 weeks, 13–14 weeks, and 15–16 weeks). (D) In contrast, the average expression levels of the late germline-specific marker genes of female FGCs in (B) in the CoV group are higher than in the Ctrl group among three gestational stages (11–12 weeks, 13–14 weeks, and 15–16 weeks). The expression level was normalized to ∼0%–100%. A larger number means a higher gene expression level. Week (W), gestational week at termination. CoV_*n* = 7, Ctrl_*n* = 5. (E and F) The percentage of expression level of all detected genes on the X chromosome compared with total genes in FGCs from the CoV and Ctrl groups. Red color scheme, female; blue color scheme, male. The bar plot is ordered by gestational age at termination, horizontally in each group. The statistical test between different groups in (E) is shown in (F). The *p* values were calculated by Wilcoxon rank-sum test. CoV_Female_*n* = 7, Ctrl_Female_*n* = 5, CoV_Male_*n* = 11, Ctrl_Male_*n* = 3. (G) The percentage of expression level of all detected genes on the Y chromosome compared with total genes in male FGCs from the CoV and Ctrl groups. The bar plot was ordered by gestational age at termination, horizontally in each group. (H) Expression level of detected genes on the Y chromosome in male FGCs. The expression level is represented as log2(FPKM+1). CoV_*n* = 11, Ctrl_*n* = 3. (I) DNA methylation level in the promoter of the genes from (H). The *p* values were calculated by Wilcoxon rank-sum test. CoV_*n* = 3, Ctrl_*n* = 3. See also Figures S2–S4.

The proper development of FGCs is regulated by a coordinated and highly dynamic group of biological processes.^14^ To further explore the specific effects of SARS-CoV-2 infection on this highly ordered developmental progress, we investigated the specific features and complex changes among dozens of representative germline marker genes. Initially, we grouped the samples by gestational stage (≤12 weeks and ≥13 weeks) and examined the expression levels of the early or the late germline marker genes. The results showed significantly higher expression of the early germline marker genes in uninfected samples in both groups. The expression pattern of the late germline marker genes was the opposite (Figures 3A and 3B). Subsequently, we divided the samples into three groups by gestational stage (11–12 weeks, 13–14 weeks, and 15–16 weeks) and compared the expression levels of these germline marker genes. The results were consistent with the two-group results (Figures 3C and 3D). These results showed that the female FGCs from the uninfected group accurately expressed the ordered developmental-stage-specific genes, as reported previously.^17^^,^^18^^,^^20^ However, this highly robust and accurately ordered expression pattern of the germ cells was altered following maternal SARS-CoV-2 infection. Specifically, in more than half of the subjects of the exposed group, early germline-specific marker genes were less expressed in female FGCs than in the uninfected group, which indicated a faster exit from pluripotency and germ cell specification^28^ (Figures 3A, 3C, and S2D). Coincidentally, late germline-specific marker genes were more highly expressed in female FGCs from the exposed group than in those in the uninfected group (Figures 3B, 3D, and S2E). Notably, these late germline-specific marker genes were mainly involved in key biological stages during female FGC development, such as mitosis, retinoid acid (RA) signaling response, meiosis, and oogenesis.^29^ In addition, the outlier performance of certain exposed samples in the PCA also echoed this phenomenon (Figure S2F). Collectively, these results suggest a trend where SARS-CoV-2 infection may result in precocious development outcomes, especially in female gonads.

Sex differentiation arises in week 9 human FGCs as the most defining event in the life cycle.^20^ Therefore, the distinct effects of maternal SARS-CoV-2 infection on FGC quality of both genders needed to be carefully explored. We first analyzed the X chromosome reactivation feature in FGCs^17^^,^^18^ and found a consistent pattern of X reactivation in female FGCs for proper human germline reprogramming following maternal SARS-CoV-2 infection (Figures 3E and 3F). Then, we explored transcriptional activity on chromosome Y (chr.Y) to obtain a better understanding of maternal infection on sex determination. Strikingly, the exposed group tended to have more active transcription activities, accompanied by a greater extent of DNA demethylation on chr.Y of FGCs than uninfected groups (Figures 3G–3I). This coincided with the above DEG analysis showing the significantly upregulated expression levels of representative Y-linked genes, including the TSPY family in male FGCs from the exposed group (Figure 2D), which further implied that infection status may contribute to the “in advance” maturation in FGC development outcomes in both genders.

### Preserved DNA demethylation pattern in human FGCs following infection

Epigenetic resetting in the early germline entails rapid DNA demethylation, which lays the foundation for subsequent gametogenesis and embryonic development in the next generation.^28^^,^^30^ Therefore, we evaluated whether such critical DNA methylome reprogramming was impaired following SARS-CoV-2 infection. Our analysis showed that the global demethylation trend was also seen in either the promoter regions or different TEs of human FGCs, when we included the surrounding somatic cells as controls. Importantly, this profound loss of DNA methylation was consistent in both exposed and uninfected groups (Figures S3A–S3E). Considering that the epigenome is extensively reprogrammed in germline development, we next focused on the impact of maternal SARS-CoV-2 infection on important epigenetic events.^17^^,^^20^ Similarly, we found that the DNA methylation in the CpG island (CGI)-containing promoters on the X chromosome and known imprinted differentially methylated regions (DMRs) was strongly erased in FGCs, while the methylation of these loci was faithfully maintained in the somatic cells in both exposed and uninfected groups without any prominent difference (Figures S4A and S4B). Together, these results suggested that FGCs could still follow the normal progression upon maternal exposure to SARS-CoV-2, including X chromosome reactivation and genomic imprint erasure. In addition, the regions that evade genome-wide DNA demethylation (referred to as “escapees”) in FGCs^20^ were also maintained following the infection (Figure S4C). Collectively, our results demonstrate that the effects of COVID-19 on DNA methylation reprogramming of FGCs are negligible, both globally and locally.

### COVID-19 induces elevated immune responses in the gonadal niche

**Figure 4:**
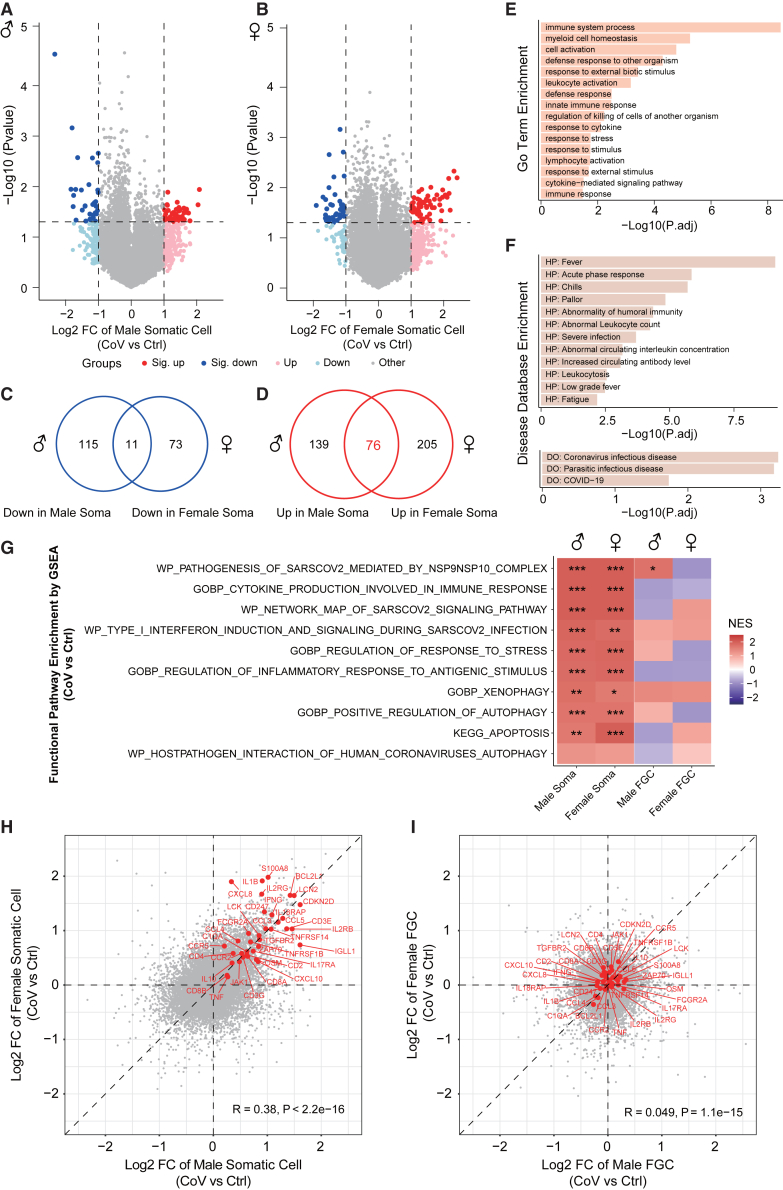
Extensive immune responses in the embryonic gonadal microenvironment preserve the integrity of FGC development following maternal SARS-CoV-2 infection (A and B) Volcano plots of DEGs identified by CoV group versus Ctrl group in male (A) or female (B) gonadal somatic cells. The alternation of gene expression is represented as log2 FC. Dark red and dark blue dots indicate genes significantly upregulated or downregulated in the CoV group (|log2 FC| > 1, *p* < 0.05; male_sig._up_*n* = 40, male_sig._down_*n* = 32, female_sig._up_*n* = 59, female_sig._down_*n* = 39). Light red and light blue dots indicate genes upregulated or downregulated in the CoV group (|log2 FC| > 1, *p* ≥ 0.05; male_up_*n* = 175, male_down_*n* = 94, female_up_*n* = 222, female_down_*n* = 45). Gray dots indicate other genes. The *p* values were calculated by Student’s t test. CoV_Male_*n* = 11, Ctrl_Male_*n* = 3, CoV_Female_*n* = 7, Ctrl_Female_*n* = 5. (C and D) Venn diagrams showing the overlap of downregulated (C) or upregulated (D) genes between male and female gonadal somatic cells. (E and F) Function annotation of the 76 co-upregulated genes in both male and female gonadal somatic cells based on the Gene Ontology (GO) database (biological processes category) (E), human Phenotype Ontology (HP) database (F, top) and human Disease Ontology (DO) database (F, bottom). The bar plots are ordered by −log10 (adjusted *p* value). (G) Gene set enrichment analysis (GSEA) of the log2 FC of the entire gene expression calculated by CoV group versus Ctrl group in male and female gonadal somatic cells and FGCs, based on the Molecular Signatures Database (MSigDB). The heatmap is ordered by normalized enrichment score (NES) of male gonadal niche cells. ∗∗∗*p* < 0.001, ∗∗*p* < 0.01, ∗*p* < 0.05. (H and I) Scatterplots showing the alternations of gene expression of all genes calculated by CoV group versus Ctrl group in male and female gonadal somatic cells (H) and FGCs (I), respectively. The alternation of gene expression is represented as log2 FC. The representative genes related to the enriched pathways shown in (E)–(G) are labeled in red. The Pearson correlation coefficients and the *p* values are shown. CoV_Male_*n* = 11, Ctrl_Male_*n* = 3, CoV_Female_*n* = 7, Ctrl_Female_*n* = 5. See also Figures S5 and S6.

The somatic cells are adjacent niches for FGCs in the gonads, which is essential for providing an appropriate microenvironment to guide specification, migration, localization to genital ridges, and sex differentiation of FGCs.^24^^,^^25^ Therefore, investigating the specific effects on surrounding somatic cells following SARS-CoV-2 infection is also of great value. Surprisingly, despite the limited changes of FGCs between the exposed and uninfected groups (Figures 2D and 2E), we discovered that SARS-CoV-2 infection specifically led to more extensive changes in gene expression of the adjacent niche cells (Figures 4A and 4B; Table S3). It is worth noting that there were more upregulated genes in niche cells from the exposed group, with higher overlap between both genders, which indicated that niche cells of both genders exhibited similar responses to the maternal exposure of SARS-CoV-2 (Figures 4C and 4D). Moreover, what greatly caught our attention was the concentrated biological processes enriched from the 76 co-upregulated genes in both male and female niche cells, including immune system process, defense response to other organism, and response to environmental stress/stimulus (Figure 4E). Despite the relatively low probability of COVID-19 invasion into the developing fetus, as reported previously,^7^^,^^31^ the gonadal niche cells from exposed groups still exhibited an extensive response to defend against the potential threat of maternal SARS-CoV-2 infection. When we mapped these genes to disease databases, symptoms related to COVID-19 infection were enriched, which indicated that the targeted responses from the surrounding somatic cells were initiated by maternal exposure to SARS-CoV-2 (Figure 4F). In addition, “coronavirus infectious disease” and “parasitic infectious disease” were also enriched, which further suggested that there may exist conservative response mechanisms against the potential threat of external pathogen invasion, such as viruses or parasites, in the developing fetus (Figure 4F).

To further decipher the effects of maternal SARS-CoV-2 infection on the somatic cells compared with uninfected groups in the developing fetus, we expanded the analysis to the entire transcriptome alternations and their functional enrichment by gene set enrichment analysis (GSEA). The immune-related pathways were also highly enriched in both male and female somatic cells from the exposed group, including innate and adaptive immunity (Figure 4G). Most of these immune responses are frequently observed following SARS-CoV-2 infection.^32^^,^^33^^,^^34^^,^^35^^,^^36^^,^^37^ For example, the induction of type I interferons and signaling is the first response leading to the innate immune reactions upon exposure to SARS-CoV-2^38^ (Figure 4G). Meanwhile, a significantly increased expression of cytokines has been discovered, including the interleukin family, chemokine family, etc., which assists with inducing an effective immune response (Figures 4G and 4H). Besides, the xenophagy process was also enriched, which indicated that adjacent niche cells employed multiple strategies to defend against the potential threat (Figure 4G). However, such a defense would compel the gonadal niche from the exposed group under higher response to extra stress (Figures 4E and 4G). Therefore, some niche cells may be sacrificed due to autophagy or apoptosis (Figure 4G). Notably, the comprehensive responses were similar between both genders, which suggested a conserved defense mechanism in the developing fetus following maternal SARS-CoV-2 injection (Figures 4G and 4H). Furthermore, it was noticed that these complex reactions following infection were only observed in the adjacent microenvironment rather than in FGCs (Figures 4G–4I and S5A–S5C).

### Maternal SARS-CoV-2 infection does not compromise the reciprocal interactions between human FGCs and gonadal niche cells

Given that reciprocal signaling communications between FGCs and their gonadal niche cells are important for FGC development,^18^ we wondered whether such extensive changes observed in gonadal niche cells would break the FGC-niche relationship. Interestingly, no prominent differences of gene expressions were detected between the exposed and uninfected groups in the typical gonadal niche interactions pathways, including bone morphogenetic protein (BMP) and NOTCH (Figures S6A–S6C). This suggested that cell communications between FGCs and the surrounding somatic cells were well maintained to support FGC development even following maternal SARS-CoV-2 infection. Considering the limited transcriptional and epigenetic changes of FGCs occurring upon SARS-CoV-2 infection, it is reasonable to hypothesize that the gonadal microenvironment was devoted to safeguarding the homeostasis of human FGCs under maternal exposure to SARS-CoV-2 by triggering an immune response only in somatic cells (Figures 4G–4I and S5A–S5C). In addition, we investigated the gene expression profiles related to the pathways vital for the maintenance and function of gonadal niche cells, including *WT1*, *NR2F2*, *TCF21*, and *PDGFRA*,^18^^,^^24^^,^^25^^,^^39^ and still found no prominent differences in this typical gene expression between the exposed and uninfected group (Figures S6D and S6E). These findings further support the trivial impact on somatic function involved in interaction pathways following maternal SARS-CoV-2 infection.

## Discussion

Due to the worldwide spread of the COVID-19 pandemic, numerous pregnant women are confronting potential threats. Whether FGCs can still retain the proper development to ensure the perpetuation of the genetic and epigenetic information across the generations and over an evolutionary timescale in such a harsh environment is an essential scientific question that urgently needs to be addressed. Here, we provided evidence that SARS-CoV-2 infection in pregnancy is primarily associated with extensive immune responses in the embryonic gonadal microenvironment, which may preserve the integrity of FGC development. However, the effects of SARS-CoV-2 infection on subsequent gamete maturation remain unclear and deserve further exploration. Intriguingly, we also noticed an unusually precocious tendency in human FGCs along with sex differentiation following maternal infection, and the elevated immune responses observed in the gonadal niche might be a possible reason (Figures 3A–3D and S5A), which requires further investigation.

Despite the adverse neonatal outcomes following maternal infection that have been reported,^4^^,^^5^^,^^6^^,^^7^ the potential effects of infection for the developing fetus remain elusive. Of note, our study focused on first-trimester human gonads, a most precious but limited-accessibility tissue derived entirely from the developing fetus that can faithfully reflect variable fetal responses against infectious diseases during early gestation. In addition, the first trimester of pregnancy is also the precise time of human primordial germ cell specification and epigenetic reprogramming. These valuable data not only provide an opportunity to explore the underlying mechanism of fetal protection against SARS-CoV-2 but also allow for a potential view on whether pregnant women during early gestation can receive COVID-19 vaccination owing to safety concerns.

Surprisingly, we observed that COVID-19, a disease not traditionally vertically transmitted (from mothers to their offspring),^40^ could trigger immune responses in the gonadal niche of the developing fetus during early gestation. Previous studies have reported that the fetus can produce immunoglobulin G (IgG) during the third trimester of gestation through natural infection or vaccination against SARS-CoV-2.^31^^,^^41^ However, whether such an anticipant immune response also occurs during early gestation remains elusive. In this study, we discovered a response to an antigenic stimulus enriched in the gonadal niche from the exposed group (Figure 4G) despite the absence of viral fragments in these samples. Meanwhile, *IGLL1*, which is important for B cells to produce antibodies for activating immune responses and clearing pathogens, was highly expressed in surrounding somatic cells from the exposed group (Figure 4H). This was consistent with the high expression levels of *FCGR2A* and *C1QA*, which can participate in the immune response by directly binding with the IgG molecule (Figure 4H). Hence, the presence of an active IgG-related pathway (a component of the coronavirus disease-COVID-19 pathway, from KEGG) in gonadal niche cells from the exposed group supported the potential for maternally derived and/or self-produced specific antibodies to provide neonatal protection from COVID-19.

Pregnancy represents a special biological state in which the protection of pregnant women against infectious diseases must be achieved, while it is also necessary to keep a balance of immunotolerance to the developing fetus. The gonadal microenvironment is assumed to be a crucial “buffer” that provides the necessary immune defense against potential threats to ensure the integrity of internal FGCs, including the proper determination and maintenance of the germ cell fate, global DNA demethylation, genomic imprint erasure, X chromosome reactivation, and epigenetic repression of retrotransposons, regardless of gender. This strategy optimizes the benefits among pregnant women, the developing fetus, and gonads in such a potential harsh environment to prepare for upcoming pandemics and guarantee the faithful transmission of genetic information to the next generation.

Whether the offspring can acquire the proper immune status during gestation to adapt to diverse external environments is important to successful human survival and reproduction. Our findings of an adaptive fetal defense in fetal gonads upon maternal SARS-CoV-2 infection provide evidence of the efficient transfer of immune memory to the next-generation individuals, which may also explain the human-specific immune innovation from an evolutionary point of view. This possible conserved mechanism, which may apply to other infectious diseases, warrants further investigation.

### Limitations of the study

Here, we provide insights into the impact of maternal SARS-CoV-2 infection on fetal germline development, but some limitations still exist. First, the relatively restricted number of COVID-19 patients enrolled in a single-center cohort needs to be considered when extrapolating the reach of our conclusions to the general population. Second, our study focused on first-trimester human gonads, which can faithfully reflect variable fetal responses against infectious diseases during early gestation. However, the effect of maternal infection on the neonate is unclear, and whether babies raised during infected pregnancy experience long COVID effects on the reproduction system needs confirmation. Third, our claims of extensive immune responses in the gonadal niche shielding FGCs from maternal infection are drawn from bulk cell analysis. Because cellular heterogeneity is a general feature of biological tissues, future studies that include single-cell sequencing technologies are recommended to better reflect how offspring can acquire the proper immune status during gestation to adapt to variable external environments.

## STAR★Methods

### Key resources table

**Table:** 

REAGENT or RESOURCE	SOURCE	IDENTIFIER
**Antibodies**		

PE mouse anti-human CD117	BD Biosciences	Cat# 555714; RRID:AB_396058, clone YB5.B8

**Critical commercial assays**		

SMARTer Stranded Total RNA-Seq Kit	Takara Bio	Cat# 634412
MethylCode Bisulfite Conversion Kit	ThermoFisher	MECOV-50
EpiArt DNA Methylation Library Kit for Illumina V3	Vazyme	NE103

**Deposited data**		

RNA-seq data	This paper	GSA: HRA005278
WGBS-seq data	This paper	GSA: HRA005277

**Software and algorithms**		

FastQC (v0.11.8)	Babraham Institute	https://www.bioinformatics.babraham.ac.uk/projects/fastqc/
Cutadapt (v2.5)	Martin^42^	http://cutadapt.readthedocs.io/en/stable/
Hisat2 (v2.1.0)	Pertea et al.^43^	http://ccb.jhu.edu/software/hisat2
SAMtools (v1.9)	Li et al.^44^	http://www.htslib.org/
featureCounts (v2.0.1)	Liao et al.^45^	http://subread.sourceforge.net/
Homer (v4.11)	Heinz et al.^46^	http://homer.ucsd.edu/homer/index.html
Bismark (v0.22.1)	Krueger and Andrews^47^	https://www.bioinformatics.babraham.ac.uk/projects/bismark/
Bedtools (v2.28.0)	Quinlan and Hall^48^	http://bedtools.readthedocs.io/en/latest/
IGV (v2.16.0)	Broad Institute	http://software.broadinstitute.org/software/igv/
R (v4.2.3)	R Core Team	https://www.r-project.org/

### Resource availability

#### Lead contact

Further information and requests for resources and reagents should be directed to and will be fulfilled by the lead contact, Jiayu Chen (chenjiayu@tongji.edu.cn).

#### Materials availability

This study did not generate new unique reagents.

#### Data and code availability

All data generated have been deposited in Genome Sequence Archive (https://bigd.big.ac.cn/gsa/) (RNA-seq data available from https://bigd.big.ac.cn/gsa-human/browse/HRA005278; WGBS-seq data available from https://bigd.big.ac.cn/gsa-human/browse/HRA005277). Accession numbers are listed in the key resources table. This study did not generate original code or algorithm. All used software tools can be found online (see key resources table). Any additional information required to reanalyze the data reported in this work paper is available from the lead contact upon request.

### Experimental model and study participant details

This study was approved by the Reproductive Study Ethics Committee of Shanghai First Maternity and Infant Hospital (KS23207). The donors in this study were pregnant women who underwent medical termination of pregnancy, did not have any other active maternal diseases except for SARS-CoV-2 infection. All of the patients signed informed consents and voluntarily donated the fetal tissues for this study.

#### Collection of human fetal samples

All human embryos from 10 to 18-week of gestation used in this study were obtained with informed consent from the donors. We collected male and female fetal gonads with intact morphology and reasonable cell viability from embryos. The stages of human embryos in this study were calculated from the last menstruation bleeding time. In total, the exposed group which refers solely to the mother testing positive for SARS-CoV-2 earlier at gestation however recovered at the termination throughout this study includes 8 female and 11 male embryos. The uninfected group referring to the mothers who were never exposed to SARS-CoV-2 includes 6 female and 5 male embryos.

Identification of externalia phenotype and genotyping were combined for sex determination of each embryo. For genotyping, the genomic DNA was lysed and used for Y chromosome genotyping (TSPY2 gene and SRY gene). The CCR6 gene on chromosome 6 was included as a control for both male and female embryos. Three primer pairs used for genotyping are listed below:

CCR6-F: 5′-GGAATATGGGGCAAAGGACA-3’.

CCR6-R: 5′-GGCTGGTTGCCTTTACTTCG-3’.

TSPY2-F: 5′-GGGCCAATGTTGTATCCTTCTC-3’.

TSPY2-R: 5′-GCCCATCGGTCACTTACACTTC-3’.

SRY-F: 5′-CCAGAAGTGAGCCTGCCTAT-3’.

SRY-R: 5′-GACTGCTTAACACGCTGCAT-3’.

### Method details

#### Isolation of human FGCs and gonadal somatic cells by FACS

Human gonads were identified and dissected under the microscopes, and were extensively washed with DPBS (plus 1% FBS) to remove any blood and other contaminants, then digested with 500 μL of Collagenase/Dispase (Sigma) at 37°C for 10–15 min. After digestion, the single-cell suspension was obtained by gently pipetting and filtered through 70 μm Pre-Separation Filters (Miltenyi Biotec) before centrifuging, discarding the supernatant and resuspending in DMEM (plus 10% FBS). According to the previous publications,^18^^,^^22^ we chose CD117 (also known as KIT) surface marker to isolate KIT-positive FGCs. Through SH800S Cell Sorter (Sony Biotechnology) sorting, we collected both the CD117-positive fraction (FGCs) and the CD117-negative fraction (FGCs and gonadal somatic cells), and the data were analyzed using FlowJo software (Tree Star). Specifically, erythrocytes in CD117-negative fraction were further removed by using red blood cell lysis buffer (Tiangen).

#### Total RNA library construction and sequencing

Isolated FGCs and gonadal somatic cells were disrupted in TRIzol Reagent (Takara), respectively and total RNAs were isolated by chloroform extraction, coupled with isopropanol precipitation with 1/10 volume of 3M NaAc and 1 μL glycogen added to the aqueous phase of each sample. RNAs were washed twice with 75% ethanol before they were eluted with nuclease-free water. Purified RNAs were then subjected to library generation using SMARTer Stranded Total RNA-Seq Kit (Takara) following the manufacturer’s instructions. Briefly, random primers were used for reverse transcription, and the amplified cDNA was then subjected to ribosomal RNA depletion. Prepared RNA-seq libraries were sequenced on the Illumina NovaSeq 6000 platform with paired ends and 150-bp read lengths at Nanjing Jiangbei New Area Biophamaceutical Public Service Platform Co., Ltd.

#### DNA extraction and PBAT library construction

Isolated cells were transferred into lysis buffer using a mouth pipette, and were then lysed for 3 h at 50°C and then heat-inactivated for 30 min at 75°C. The released genomic DNA, together with 1% unmethylated lambda DNA (Thermo Scientific), were sheared into 150 bp to 350 bp by Covaris S2 before subjecting to bisulfite conversion using a MethylCode Bisulfite Conversion Kit (Invitrogen) according to the manufacturer’s instructions. The bisulfite-converted templates were then used to prepare the sequencing library through EpiArt DNA Methylation Library Kit following the manufacturer’s instructions. The PBAT libraries were also sequenced on Illumina NovaSeq with paired ends and 150-bp read lengths at Nanjing Jiangbei New Area Biophamaceutical Public Service Platform Co., Ltd.

#### Processing of RNA-seq data

FastQC (v0.11.8) was used to check the quality of raw sequencing data. Low-quality and adaptor sequences were trimmed from the reads using Cutadapt (v2.5)^42^ with parameters: -a AGATCGGAAGAGC -AAGATCGGAAGAGC --trim-n -m 75 -q 20,20. Then, the reads were mapped to the human genome (hg19, obtained from UCSC) using Hisat2 (v2.1.0)^43^ with parameters: --dta --no-discordant --no-mixed --no-unal. The SAMtools (v1.9)^44^ was used to transfer the mapping results from sam format to position sorted bam format. After that, Mapped reads were subsequently assembled into transcripts guided by the UCSC GTF annotation files (hg19) using featureCounts (v2.0.1)^45^ with parameters: -M -p -B -C. Finally, the expression level of each gene was quantified as FPKM (fragments per kilobase of transcript sequence per million read pairs mapped) based on featureCounts raw outcome in R (v4.2.3). The gene expression is transformed to log2(FPKM+1) and quantile normalized by preprocessCore (v1.60.2) package in R. The function annotation of genes (including GO/HP/DO enrichment and GSEA) were performed by clusterProfiler (v4.6.2) package in R.

The expression at human repetitive elements were quantified using Homer (v4.11).^46^ In brief, mapped data were processed with the makeTagDirectory command in Homer with the parameter “-keepOne”. The tag files of samples were analyzed using the “analyzeRepeats.pl repeats” command in Homer with RPKM normalization. For the analysis of repeat classes, the "-condenseL1" parameter was used to combine the read counts of repeats with the same class annotation.

#### Processing of WGBS-seq data

FastQC (v0.11.8) was used to check the quality of raw sequencing data. Low-quality and adaptor sequences were trimmed from the reads using Cutadapt (v2.5)^42^ with parameters: -a AGATCGGAAGAGC -AAGATCGGAAGAGC --trim-n -m 50 -q 20,20 -U 10. Then, the clean reads were mapped to the human genome (hg19, obtained from UCSC) using Bismark (v0.22.1)^47^ with parameters: -N 1 -X 500. DNA methylation level of lambda was used to check the bisulfite conversion efficiency. Then, the unmapped reads, non-uniquely mapped reads, and PCR duplicates were removed by deduplicate_bismark from Bismark with default parameters. Next, the methylation sites were called and the DNA methylation level for individual CpG sites were calculated by the bismark_methylation_extractor from Bismark. Only the CpG sites that were covered by at least three reads were kept for all subsequent analyses. Additionally, the bedgraph files were converted to bigwig files. Integrative Genomic Viewer (IGV, v2.16.0) was used for the visualization of DNA methylation profiles.

The methylation level in a region was computed as the average of the methylation percentages of all detected CpG sites in the region by Bedtools (v2.28.0),^48^ and the regions containing at least five CpG sites were kept. The genome regions involved in this analysis part including promoter (defined as the 2Kb region upstream to TSS and 1Kb region downstream to TSS), CpG islands (obtained from UCSC hg19), and repeat elements (LINE, SILE, LTR, SVA and Satellite, obtained from UCSC hg19).

### Quantification and statistical analysis

For high-throughput sequencing, gene expression values were log2 transformed and normalized. Data analysis was performed by R. All statistical tests were comprehensively and clearly illustrated in the parallel figure legends. Two-tailed unpaired *t*-test or Wilcoxon rank-sum test was used for the comparison between the two groups. Pearson test was used for correlation analysis. Error bars in the graphical data represent the Standard Error of the Mean (SEM). The boxplots showing the distribution of the data. The boxes indicate the 25th to 75th percentiles. The horizontal lines within the boxes indicate the median levels. The whiskers indicate 1.5×the inter-quartile range. p-value <0.05 was considered to indicate statistical significance for all analyses.

## Supplemental information

Document S1. Figures S1–S6

Table S1. Summary of datasets generated in this study, related to Figure 1

Table S2. DEGs identified by female versus male gonadal somatic cells in the Ctrl group, related to Figure 2

Table S3. DEGs identified by the CoV group versus Ctrl group in male and female FGCs and gonadal somatic cells, related to Figures 2 and 4

Document S2. Article plus supplemental information
