# Supplementary material for: The gonadal niche safeguards human fetal germline cell development following maternal SARS-CoV-2 infection
Source: Cell Rep Med. 2024 Apr 16;5(5):101515. doi: 10.1016/j.xcrm.2024.101515 (PMC11148563; doi:10.1016/j.xcrm.2024.101515)
Supplement: Document S1. Figures S1–S6 [file mmc1.pdf]

Cell Reports Medicine, Volume *5*

Supplemental information

The gonadal niche safeguards human fetal germline cell development following maternal SARS-CoV-2 infection

Shijun Shen, Mengting Wang, Xiaocui Li, Beiying Wang, Wei Hong, Wei Li, Ben Xu, Zhenxiang Guo, Ruichen Han, Shanru Yi, Zhiping Wu, Xiaoying He, Liping Wang, Qianshu Zhu, Guang Yang, Hong Wang, Qiaolin Deng, Jiayu Chen, Shaorong Gao, Cizhong Jiang, and Rui Gao

Document S1. Figure S1-S6, related to Figure 1-4.

Figure S1. Isolation of human FGCs and gonadal somatic cells by FACS following maternal SARS-CoV-2 infection, related to Figure 1.

Figure S2. Expression pattern of human FGCs following maternal SARS-CoV-2 infection, related to Figure 2, 3.

Figure S3. DNA methylation pattern of human FGCs and gonadal somatic cells following maternal SARS-CoV-2 infection, related to Figure 2, 3.

Figure S4. DNA methylation reprogramming faithfully maintained in human FGCs following maternal SARS-CoV-2 infection, related to Figure 3.

Figure S5. Extensive immune responses in gonadal niche shield FGCs from maternal SARS-CoV-2 infection, related to Figure 4.

Figure S6. Human FGC-niche communication was not compromised following maternal SARS-CoV-2 infection, related to Figure 4.

A

Patient ID	Maternal SARS-CoV-2 status	Gender	Gestational age at infection	Gestational age at termination	hFGC		Soma	
					Total RNA-seq	WGBS-seq	Total RNA-seq	WGBS-seq
P1	Maternal exposed (CoV)	Female	4W	11W	√		√	
P2	Maternal exposed (CoV)	Female	3W	11W	√		√	
P3	Maternal exposed (CoV)	Female	3W	11W	√		√	
P4	Maternal exposed (CoV)	Female	4D	12W	√		√	
P5	Maternal exposed (CoV)	Female	5W	13W	√		√	
P6	Maternal exposed (CoV)	Female	2W	13W	√	√	√	√
P7	Maternal exposed (CoV)	Female	5W	15W	√		√	
P8	Uninfected (Ctrl)	Female	N/A	11W	√		√	
P9	Uninfected (Ctrl)	Female	N/A	11W	√		√	
P10	Uninfected (Ctrl)	Female	N/A	12W	√		√	
P11	Uninfected (Ctrl)	Female	N/A	14W	√		√	
P12	Uninfected (Ctrl)	Female	N/A	16W	√		√	
P13	Maternal exposed (CoV)	Male	4W	10W	√		√	
P14	Maternal exposed (CoV)	Male	2W	11W	√		√	
P15	Maternal exposed (CoV)	Male	4W	11W	√		√	
P16	Maternal exposed (CoV)	Male	7W	11W	√		√	
P17	Maternal exposed (CoV)	Male	3W	12W	√	√	√	√
P18	Maternal exposed (CoV)	Male	2W	12W	√	√	√	√
P19	Maternal exposed (CoV)	Male	5W	13W	√		√	
P20	Maternal exposed (CoV)	Male	5W	14W	√		√	
P21	Maternal exposed (CoV)	Male	5W	15W	√		√	
P22	Maternal exposed (CoV)	Male	2W	15W	√		√	
P23	Maternal exposed (CoV)	Male	9W	17W	√		√	
P24	Uninfected (Ctrl)	Male	N/A	10W	√		√	
P25	Uninfected (Ctrl)	Male	N/A	12W	√		√	
P26	Uninfected (Ctrl)	Male	N/A	12W	√		√	
P27	Uninfected (Ctrl)	Male	N/A	11W		√		√
P28	Uninfected (Ctrl)	Male	N/A	11W		√		√
P29	Uninfected (Ctrl)	Female	N/A	13W		√		√

D, day. W, week. N/A, not available.

B

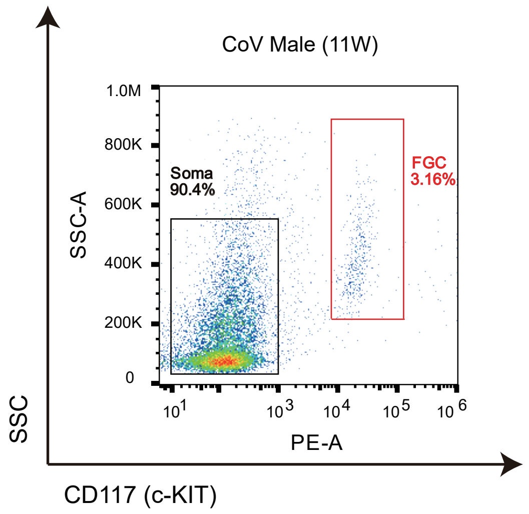

C

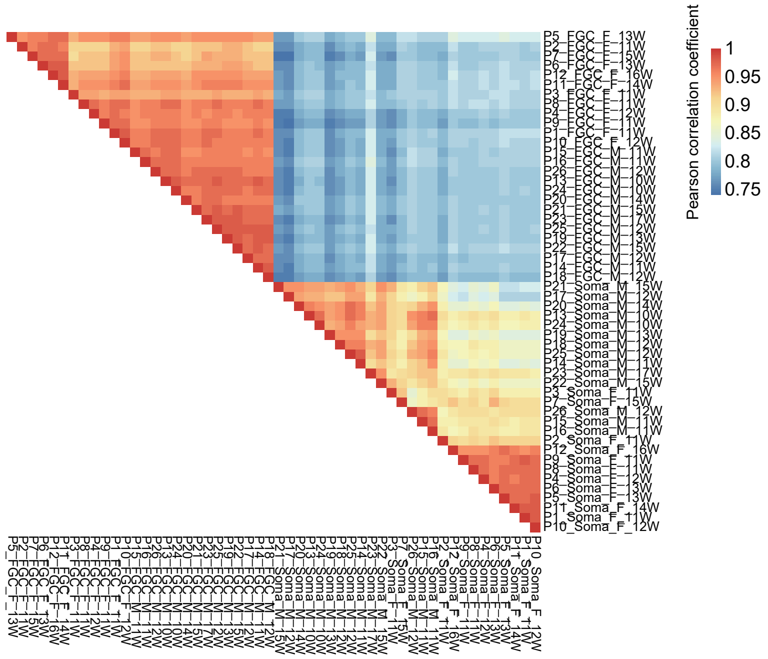

Figure S1

Figure S1. Isolation of human FGCs and gonadal somatic cells by FACS following maternal SARS-CoV-2 infection, related to Figure 1.

(A) Details of samples collected from developing fetuses for total RNA-seq or WGBS-seq. D, day. W, week. N/A, not available.

(B) Representative FACS images from one 11W male gonad following maternal infection. W, gestational week at termination.

(C) Heatmap showing Pearson correlation coefficients of all transcriptomes. F, female. M, male. W, gestational week at termination.

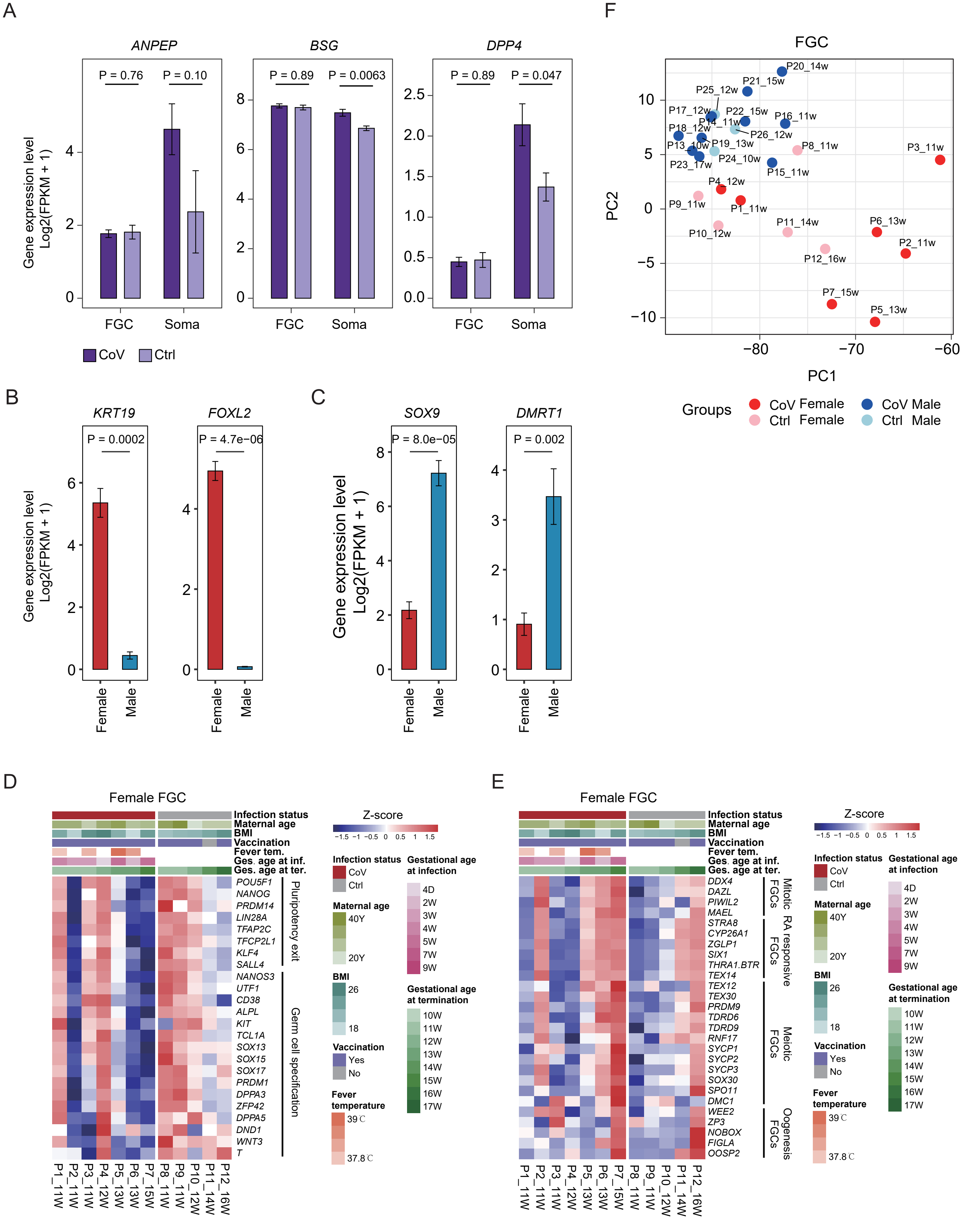
A
ANPEP
BSG
DPP4
P = 0.76
P = 0.10
P = 0.89
P = 0.0063
P = 0.89
P = 0.047
Gene expression level
Log2(FPKM + 1)
FGC
Soma
CoV
Ctrl
B
KRT19
FOXL2
P = 0.0002
P = 4.7e−06
Female
Male
C
SOX9
DMRT1
P = 8.0e−05
P = 0.002
D
Female FGC
Infection status
Maternal age
BMI
Vaccination
Fever tem.
Ges. age at inf.
Ges. age at ter.
POU5F1
NANOG
PRDM14
LIN28A
TFAP2C
TFCP2L1
KLF4
SALL4
NANOS3
UTF1
CD38
ALPL
KIT
TCL1A
SOX13
SOX15
SOX17
PRDM1
DPPA3
ZFP42
DPPA5
DND1
WNT3
T
Pluripotency exit
Germ cell specification
P1_11W
P2_11W
P3_11W
P4_12W
P5_13W
P6_13W
P7_15W
P8_11W
P9_11W
P10_12W
P11_14W
P12_16W
Z-score
Infection status
CoV
Ctrl
Maternal age
40Y
20Y
BMI
26
18
Vaccination
Yes
No
Fever temperature
39 ℃
37.8 ℃
Gestational age at infection
4D
2W
3W
4W
5W
7W
9W
Gestational age at termination
10W
11W
12W
13W
14W
15W
16W
17W
E
Female FGC
DDX4
DAZL
PIWIL2
MAEL
STRA8
CYP26A1
ZGLP1
SIX1
THRA1.BTR
TEX14
TEX12
TEX30
PRDM9
TDRD6
TDRD9
RNF17
SYCP1
SYCP2
SYCP3
SOX30
SPO11
DMC1
WEE2
ZP3
NOBOX
FIGLA
OOSP2
Mitotic FGCs
RA responsive FGCs
Meiotic FGCs
Oogenesis FGCs
F
FGC
PC1
PC2
Groups
CoV Female
Ctrl Female
CoV Male
Ctrl Male

Figure S2

Figure S2. Expression pattern of human FGCs following maternal SARS-CoV-2 infection, related to Figure 2, 3.

(A) Expression level of selected SCARFs (SARS-CoV-2- and coronavirus-associated receptors and factors) in FGCs and gonadal somatic cells. The expression level was represented as log2(FPKM+1). P values were calculated by Wilcoxon rank sum test. CoV_n = 18, Ctrl_n = 8.

(B and C) Bar plots showing expression level of selected female (B) and male (C) gonadal somatic marker genes. The expression level was represented as log2(FPKM+1). The Student's *t*-test was performed to examine the statistical difference between female and male in Ctrl group. Red color scheme, female. Blue color scheme, male. Ctrl_Female_n = 5, Ctrl_Male_n = 3.

(D and E) Comparison of the expression level of early (D) or late (E) germline specific marker genes of female FGCs between CoV and Ctrl group. The expression level was Z-scored. The clinical characteristics of these samples were shown above correspondingly. Y, year. W, week. D, day. The heatmaps were ordered by gestational age at termination horizontally in each infection status.

(F) Principal component analysis (PCA) of the transcriptome of FGCs from CoV and Ctrl group. Red color scheme, female. Blue color scheme, male. W, gestational week at termination.

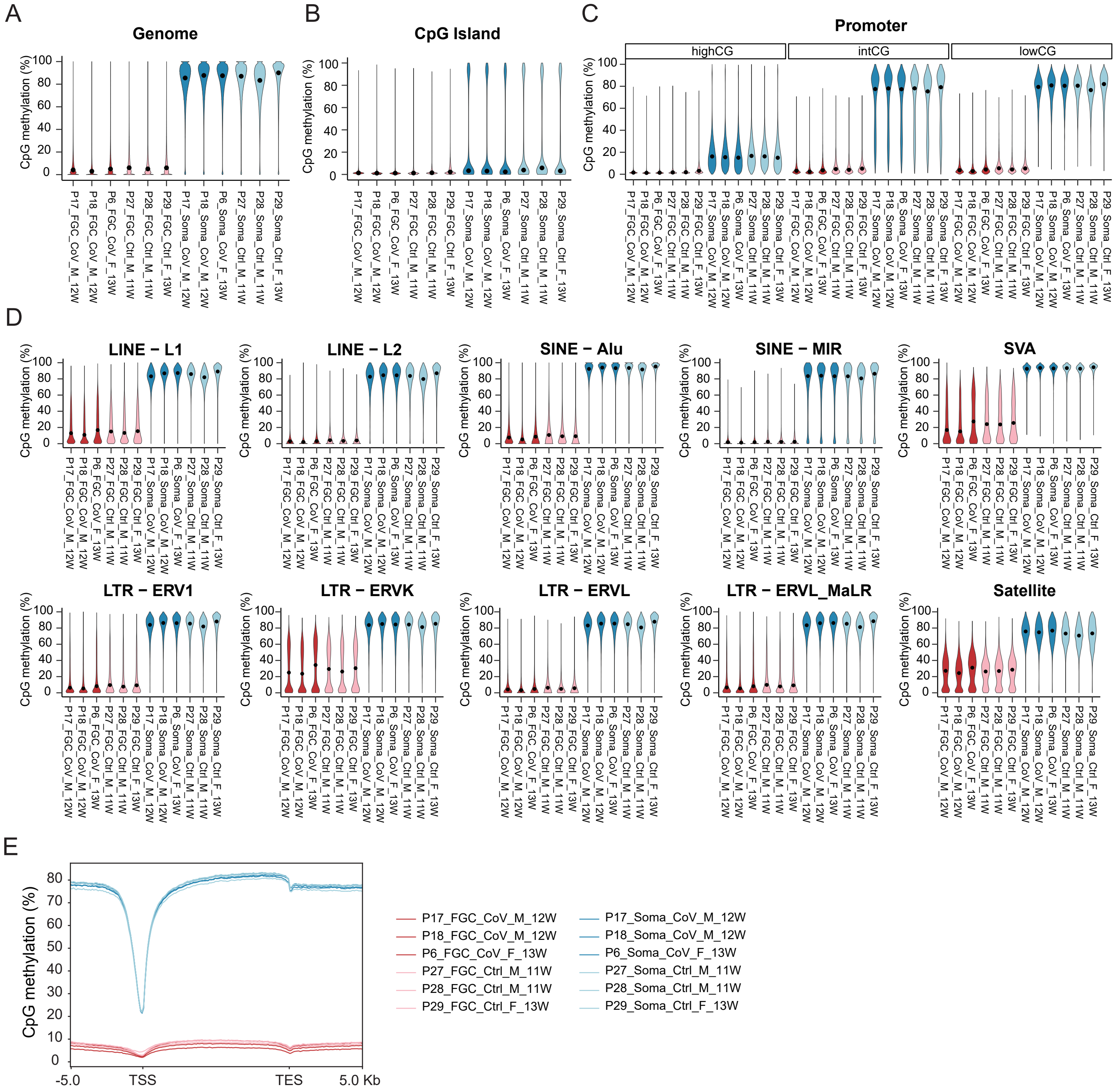
A
Genome
B
CpG Island
C
Promoter
highCG
intCG
lowCG
D
LINE − L1
LINE − L2
SINE − Alu
SINE − MIR
SVA
LTR − ERV1
LTR − ERVK
LTR − ERVL
LTR − ERVL_MaLR
Satellite
CpG methylation (%)
P17_FGC_CoV_M_12W
P18_FGC_CoV_M_12W
P6_FGC_CoV_F_13W
P27_FGC_Ctrl_M_11W
P28_FGC_Ctrl_M_11W
P29_FGC_Ctrl_F_13W
P17_Soma_CoV_M_12W
P18_Soma_CoV_M_12W
P6_Soma_CoV_F_13W
P27_Soma_Ctrl_M_11W
P28_Soma_Ctrl_M_11W
P29_Soma_Ctrl_F_13W
E
-5.0
TSS
TES
5.0 Kb

Figure S3

Figure S3. DNA methylation pattern of human FGCs and gonadal somatic cells following maternal SARS-CoV-2 infection, related to Figure 2, 3.

(A - D) Violin plots showing distribution of CpG methylation level in 500 bp sliding windows of whole genome (A), CpG Islands (B), promoter of genes (C), major human repetitive element classes and families (D) of FGCs and gonadal somatic cells from CoV and Ctrl group. F, female. M, male. W, gestational week at termination.

(E) Averaged CpG methylation level profiles of all genes from 5 Kb upstream (-) of transcription start sites (TSSs), through scaled gene bodies to 5 Kb downstream (+) of transcription end sites (TESs) in FGCs and gonadal somatic cells from CoV and Ctrl group. F, female. M, male. W, gestational week at termination.

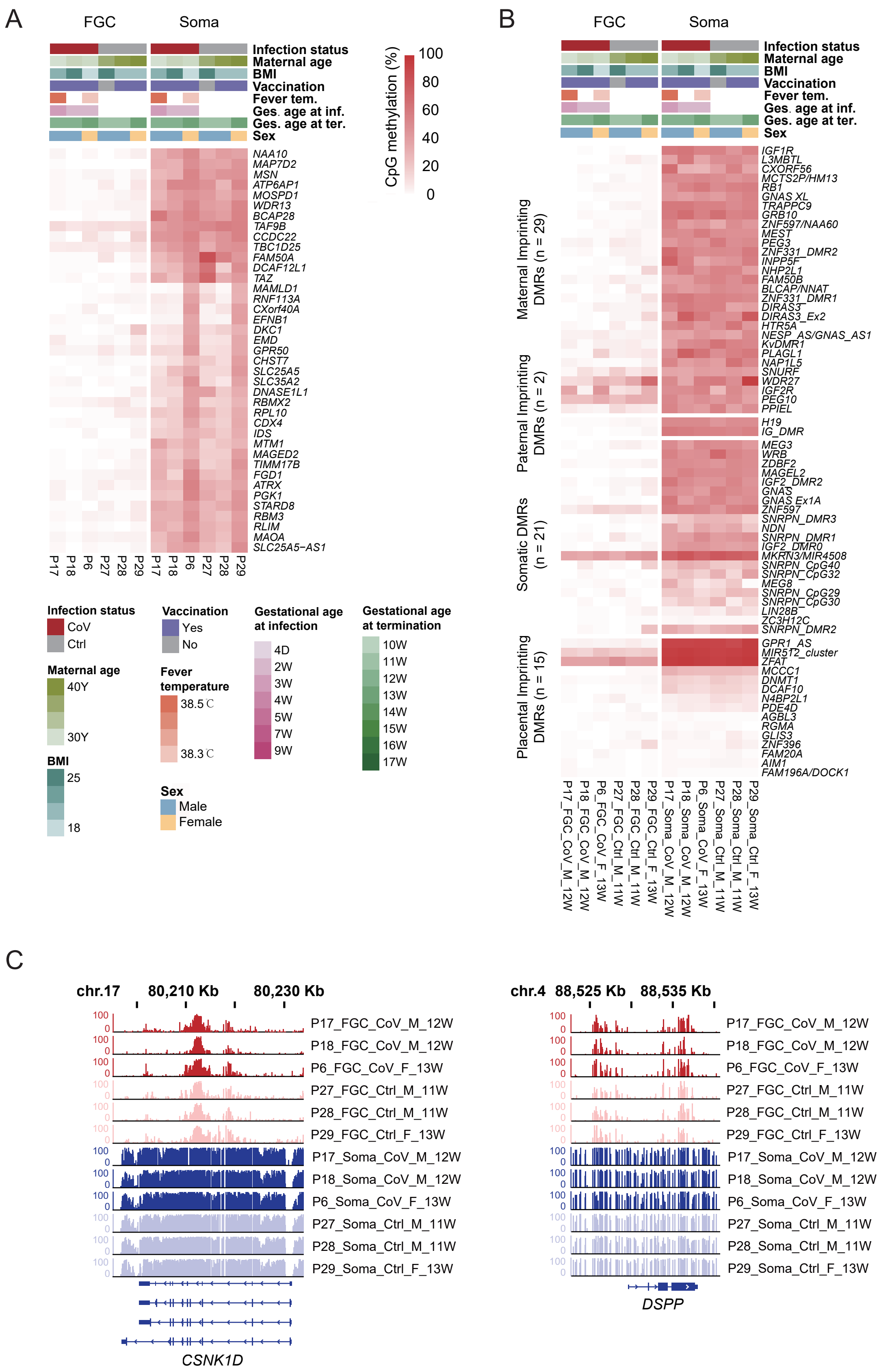

A
FGC
Soma
Infection status
Maternal age
BMI
Vaccination
Fever tem.
Ges. age at inf.
Ges. age at ter.
Sex
CpG methylation (%)
NAA10
MAP7D2
MSN
ATP6AP1
MOSPD1
WDR13
BCAP28
TAF9B
CCDC22
TBC1D25
FAM50A
DCAF12L1
TAZ
MAMLD1
RNF113A
CXorf40A
EFNB1
DKC1
EMD
GPR50
CHST7
SLC25A5
SLC35A2
DNASE1L1
RBMX2
RPL10
CDX4
IDS
MTM1
MAGED2
TIMM17B
FGD1
ATRX
PGK1
STARD8
RBM3
RLIM
MAOA
SLC25A5-AS1
P17
P18
P6
P27
P28
P29
Infection status
CoV
Ctrl
Maternal age
40Y
30Y
BMI
25
18
Vaccination
Yes
No
Fever temperature
38.5℃
38.3℃
Sex
Male
Female
Gestational age at infection
4D
2W
3W
4W
5W
7W
9W
Gestational age at termination
10W
11W
12W
13W
14W
15W
16W
17W
B
Maternal Imprinting DMRs (n = 29)
Paternal Imprinting DMRs (n = 2)
Somatic DMRs (n = 21)
Placental Imprinting DMRs (n = 15)
IGF1R
L3MBTL
CXORF56
MCTS2P/HM13
RB1
GNAS XL
TRAPPC9
GRB10
ZNF597/NAA60
MEST
PEG3
ZNF331_DMR2
INPP5F
NHP2L1
FAM50B
BLCAP/NNAT
ZNF331_DMR1
DIRAS3
DIRAS3_Ex2
HTR5A
NESP_AS/GNAS_AS1
KvDMR1
PLAGL1
NAP1L5
SNURF
WDR27
IGF2R
PEG10
PPIEL
H19
IG_DMR
MEG3
WRB
ZDBF2
MAGEL2
IGF2_DMR2
GNAS
GNAS Ex1A
ZNF597
SNRPN_DMR3
NDN
SNRPN_DMR1
IGF2_DMR0
MKRN3/MIR4508
SNRPN_CpG40
SNRPN_CpG32
MEG8
SNRPN_CpG29
SNRPN_CpG30
LIN28B
ZC3H12C
SNRPN_DMR2
GPR1_AS
MIR512_cluster
ZFAT
MCCC1
DNMT1
DCAF10
N4BP2L1
PDE4D
AGBL3
RGMA
GLIS3
ZNF396
FAM20A
AIM1
FAM196A/DOCK1
P17_FGC_CoV_M_12W
P18_FGC_CoV_M_12W
P6_FGC_CoV_F_13W
P27_FGC_Ctrl_M_11W
P28_FGC_Ctrl_M_11W
P29_FGC_Ctrl_F_13W
P17_Soma_CoV_M_12W
P18_Soma_CoV_M_12W
P6_Soma_CoV_F_13W
P27_Soma_Ctrl_M_11W
P28_Soma_Ctrl_M_11W
P29_Soma_Ctrl_F_13W
C
chr.17
80,210 Kb
80,230 Kb
CSNK1D
chr.4
88,525 Kb
88,535 Kb
DSPP

Figure S4

Figure S4. DNA methylation reprogramming faithfully maintained in human FGCs following maternal SARS-CoV-2 infection, related to Figure 3.

(A and B) DNA methylation dynamics of CGI-containing promoters on X chromosome (A) or imprinted differentially methylated regions (DMRs) (B) of FGCs and gonadal somatic cells from CoV and Ctrl group. The clinical characteristics of these samples were shown above correspondingly. Y, year. W, week. D, day. The heatmaps were ordered by gestational age at termination horizontally in each infection status.

(C) IGV plots showing the DNA methylation level profiles of two representative regions that evaded genome-wide DNA demethylation in FGCs (“escapees”). F, female. M, male. W, gestational week at termination.

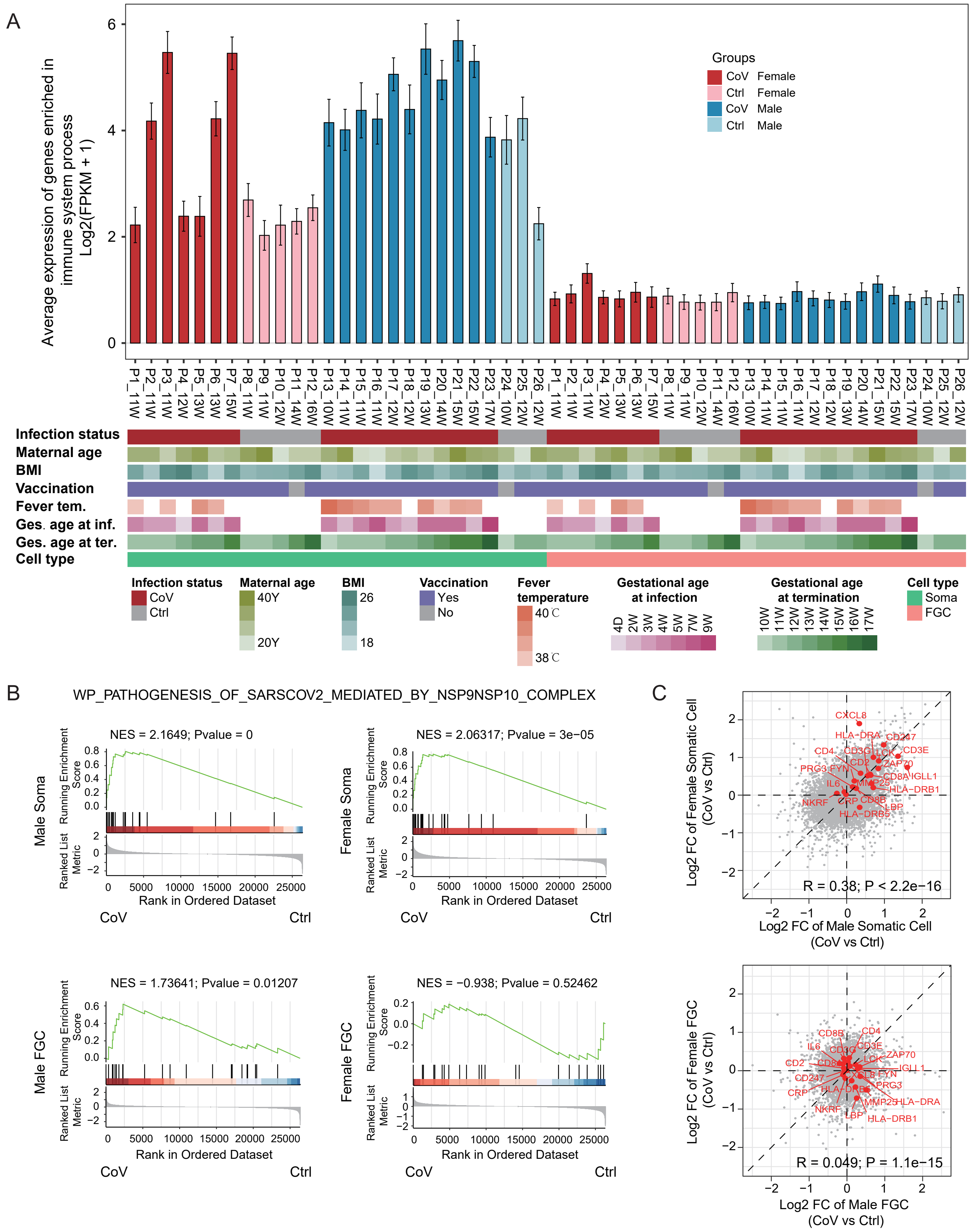
A
Average expression of genes enriched in immune system process Log2(FPKM + 1)
Groups
CoV Female
Ctrl Female
CoV Male
Ctrl Male
Infection status
Maternal age
BMI
Vaccination
Fever tem.
Ges. age at inf.
Ges. age at ter.
Cell type
Infection status
CoV
Ctrl
Maternal age
40Y
20Y
BMI
26
18
Vaccination
Yes
No
Fever temperature
40 ℃
38 ℃
Gestational age at infection
4D 2W 3W 4W 5W 7W 9W
Gestational age at termination
10W 11W 12W 13W 14W 15W 16W 17W
Cell type
Soma
FGC
B
WP_PATHOGENESIS_OF_SARSCOV2_MEDIATED_BY_NSP9NSP10_COMPLEX
Male Soma
NES = 2.1649; Pvalue = 0
Female Soma
NES = 2.06317; Pvalue = 3e−05
Male FGC
NES = 1.73641; Pvalue = 0.01207
Female FGC
NES = −0.938; Pvalue = 0.52462
Running Enrichment Score
Ranked List Metric
Rank in Ordered Dataset
CoV
Ctrl
C
Log2 FC of Female Somatic Cell (CoV vs Ctrl)
Log2 FC of Male Somatic Cell (CoV vs Ctrl)
R = 0.38; P < 2.2e−16
Log2 FC of Female FGC (CoV vs Ctrl)
Log2 FC of Male FGC (CoV vs Ctrl)
R = 0.049; P = 1.1e−15

Figure S5

Figure S5. Extensive immune responses in gonadal niche shield FGCs from maternal SARS-CoV-2 infection, related to Figure 4.

(A) The average expression level of co-upregulated genes in both male and female gonadal somatic cells and FGCs, which enriched in the top GO term “immune system process”. The clinical characteristics of these samples were shown below correspondingly. Y, year. W, week. D, day. The bar plot was ordered by gestational age at termination horizontally in each group.

(B) The selected top enriched pathway of Figure 4G was shown by GSEA enrichment plot in male and female gonadal somatic cells and FGCs, respectively. The NES and P value were shown. NES, normalized enrichment score.

(C) Scatter plots showing the alternations of gene expression of all genes calculated by CoV group versus Ctrl group in male and female gonadal somatic cells (C, top) and FGCs (C, bottom), respectively. The alternation of gene expression was represented as log2 fold change (log2 FC). The representative genes related to the pathways shown in (B) were labeled in red. The Pearson correlation coefficients and the P values were shown. CoV_Male_n = 11, Ctrl_Male_n = 3, CoV_Female_n = 7, Ctrl_Female_n = 5.

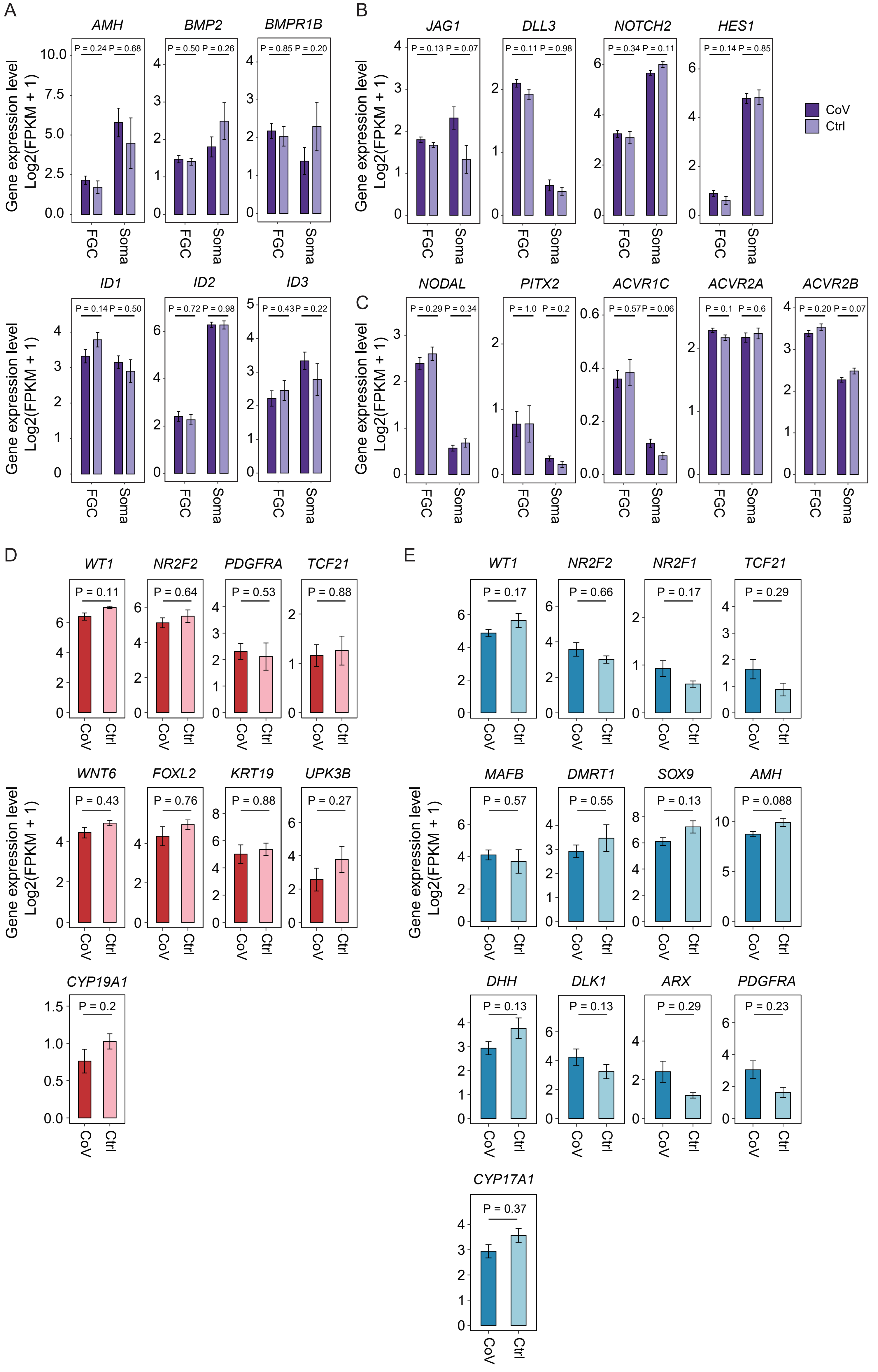
A
B
C
D
E
AMH
BMP2
BMPR1B
JAG1
DLL3
NOTCH2
HES1
ID1
ID2
ID3
NODAL
PITX2
ACVR1C
ACVR2A
ACVR2B
CoV
Ctrl
FGC
Soma
Gene expression level
Log2(FPKM + 1)
P = 0.24 P = 0.68
P = 0.50 P = 0.26
P = 0.85 P = 0.20
P = 0.13 P = 0.07
P = 0.11 P = 0.98
P = 0.34 P = 0.11
P = 0.14 P = 0.85
P = 0.14 P = 0.50
P = 0.72 P = 0.98
P = 0.43 P = 0.22
P = 0.29 P = 0.34
P = 1.0 P = 0.2
P = 0.57 P = 0.06
P = 0.1 P = 0.6
P = 0.20 P = 0.07
WT1
NR2F2
PDGFRA
TCF21
P = 0.11
P = 0.64
P = 0.53
P = 0.88
WNT6
FOXL2
KRT19
UPK3B
P = 0.43
P = 0.76
P = 0.88
P = 0.27
CYP19A1
P = 0.2
WT1
NR2F2
NR2F1
TCF21
P = 0.17
P = 0.66
P = 0.17
P = 0.29
MAFB
DMRT1
SOX9
AMH
P = 0.57
P = 0.55
P = 0.13
P = 0.088
DHH
DLK1
ARX
PDGFRA
P = 0.13
P = 0.13
P = 0.29
P = 0.23
CYP17A1
P = 0.37

Figure S6

Figure S6. Human FGC-niche communication was not compromised following maternal SARS-CoV-2 infection, related to Figure 4.

(A - C) Bar plots showing expression level of the specific ligands, receptors, and target genes of the BMP (A), NOTCH (B) and NODAL (C) signaling pathways. The expression level was represented as log2(FPKM+1). P values were calculated by Wilcoxon rank sum test. CoV_n = 18, Ctrl_n = 8.

(D and E) Bar plots showing expression level of selected female (D) and male (E) gonadal somatic marker genes. The expression level was represented as log2(FPKM+1). The Student's *t*-test was performed to examine the statistical difference between the CoV group and Ctrl group. Red color scheme, female. Blue color scheme, male. CoV_Female_n = 7, Ctrl_Female_n = 5, CoV_Male_n = 11, Ctrl_Male_n = 3.
